# Dietary intake of individuals receiving Supplemental Nutrition Assistance Program and food pantry assistance in North Texas

**DOI:** 10.1017/S136898002200074X

**Published:** 2023-05

**Authors:** Seema Jain, Kathryn Shahan, Michael Bowen, Sandi L Pruitt

**Affiliations:** 1 Internal Medicine, The University of Texas Southwestern Medical Center, 5323 Harry Hines Blvd, E1, Dallas, TX 75390, USA; 2 Population and Data Sciences, The University of Texas Southwestern Medical Center, Dallas, TX, USA; 3 Harold C. Simmons Cancer Center, The University of Texas Southwestern Medical Center, Dallas, TX, USA

**Keywords:** Food assistance, Supplemental Nutrition Assistance Program, Dietary intake, Food pantries

## Abstract

**Objective:**

Food pantries and the Supplemental Nutrition Assistance Program (SNAP) are widely available resources for individuals facing food insecurity, yet the dietary quality of individuals using both programmes is not well characterised. We describe the dietary intake of individuals in North Texas who use both food pantries and SNAP to identify nutritional gaps and opportunities to improve food assistance programmes.

**Design:**

We analysed baseline data from a randomised controlled trial examining food security and dietary intake. At baseline, we administered the validated, 26-item Dietary Screener Questionnaire (DSQ). We calculated descriptive statistics for dietary intake variables and compared with the 2020–2025 Dietary Guidelines for Americans recommended intake values.

**Setting:**

Two large food pantries in Dallas County, TX.

**Participants:**

Eligible participants were English or Spanish speaking adults receiving SNAP benefits who had used the food pantry within the last 4 months.

**Results:**

We analysed baseline DSQ data from 320 participants (mean age 47 years; 90% female; 45% Black or African American; 37% Hispanic or Latino). Despite receiving SNAP benefits and food pantry assistance, most participants did not meet the minimum recommended intake values for fruits (88.4%), vegetables (97.4%), fibre (90·7%), whole grains (99·7%), dairy products (98·4%) and Ca (83·4%). Furthermore, 73·2% of participants exceeded the maximum recommended intake for added sugar. Still, the gap between median daily intake and recommended daily intake could be partially bridged with food obtained through current food assistance programmes.

**Conclusions:**

Multilevel, coordinated approaches within both SNAP and food pantry networks are needed to improve diet quality in individuals receiving food assistance.

Food assistance in the USA is obtained through public governmental food assistance programmes and private charitable food assistance programmes. The largest US governmental food assistance programme is Supplemental Nutrition Assistance Program (SNAP), which provides monthly benefit allotments based on household income and household size that can be used at participating retailers to purchase food, non-alcoholic beverages, and seeds and plants that produce food^(1)^. In 2019, SNAP served 38 million people nationwide^(2)^. The charitable food assistance system consists predominantly of networks of food banks, which distribute food to non-profit partner agencies including food pantries, kitchens and shelters^(1)^. In 2014, Feeding America, the largest network of food banks in the USA, served 46·5 million people.^(1)^

Public and private food assistance programmes help alleviate food insecurity^(3)^, a key social determinant of health defined as the lack of access to enough food for all members of a household to live healthy, active lives^(4)^. Still, our knowledge of the dietary quality of clients who use these programmes remains limited. While most Americans do not meet national dietary recommendations, the diet quality of food pantry clients is even worse than that of the general population^(5,6)^. Findings regarding SNAP participants are mixed, suggesting this population has a lower dietary quality compared with higher-income non-participants but likely no significant differences in macronutrient or micronutrient intake compared with income-eligible non-participants^(7)^. Furthermore, while regional studies on food pantry clients indicate that frequency of food pantry use and food security may be positively associated with diet quality^(8,9)^, the association of household size with diet quality in this population is unknown. Given the theoretical impact that household size has on eating patterns and availability of food, this is a metric of interest.

Previous studies on diet quality of clients receiving food assistance have typically focused on one type of food assistance programme in isolation^(3–9)^. However, clients often receive more than one type of food assistance; nationally, 45% of charitable food assistance clients also participate in SNAP^(3)^. Moreover, individuals receiving both public and private food assistance have worse health and increased prevalence of diet-related chronic disease compared with those receiving only one type of food assistance or no food assistance^(10)^. Given overwhelming evidence indicating that a healthy dietary pattern can help prevent and manage chronic disease^(11)^, the dietary quality of the millions of individuals accessing both public and private food assistance programmes is of public health concern.

We sought to understand the dietary intake of food pantry clients who were also receiving SNAP benefits in Dallas County, Texas. We conducted this study to identify nutritional gaps and outline future opportunities for food assistance programmes to improve diet quality. Additionally, to inform the need for interventions targeted to subsets of clients, we explored whether dietary intake differed by socio-demographic characteristics.

## Methods

We analysed baseline data from the Supplemental Nutrition Assistance Program Appointment Coordination (SNAP-AC) randomised controlled trial. SNAP-AC was conducted at two large food pantries in Dallas County, Texas and examined the effectiveness of coordinating pantry visits and SNAP benefit distributions to improve client nutrition, food security and self-rated health^(12)^. The Dallas-Fort Worth metroplex in North Texas is the fourth largest in the nation and the two food pantries studied are high capacity; they each provided more than 11 million pounds of food in 2020 (email, J. Taylor and J. Kramer, December 2021). Both food pantries were client choice at the time of enrolment and sourced foods (including frozen meat, fresh dairy products, fresh produce, canned items, whole grains and snacks) based on the North Texas Food Bank’s Food and Nutrition Policy (email, M. Charlot, July 2021). Households could receive food from the pantries 1–2 times per month. Enrolled participants were English or Spanish speaking adults, receiving SNAP benefits and had visited the food pantry within 4 months prior to enrolment. SNAP-AC enrolled 327 participants between October 2019 and March 2020. In this analysis of SNAP-AC baseline data, we included 320 participants after excluding those with missing or incomplete baseline data.

Food pantries collected socio-demographic data at enrolment, including age, race and ethnicity, sex, marital status, income level, educational attainment, household size, age of household members and food security status. Monthly household income level was expressed as a fraction of the 2019 federal poverty level^(13)^. Food security in the past month was assessed using the US Department of Agriculture’s 10-item US Adult Food Security Survey Module^(14)^. In this measure, the sum of affirmative responses to the ten questions creates a raw score, which is categorised as high, marginal, low or very low food security^(14)^. High and marginal food security are classified as food secure, and low and very low food security are classified as food insecure^(14)^. Baseline dietary information was collected using the Dietary Screener Questionnaire (DSQ), a 26-item validated survey assessing frequency of select food and beverage consumption in the past month to estimate daily intake of seven food and nutrient categories: fruits, vegetables, whole grains, dairy products, fibre, Ca and added sugar^(15)^. The DSQ was included in the National Health and Nutrition Examination Survey (NHANES) 2009–2010 and calibrated against two non-consecutive 24-h recalls^(15)^. In our study, the DSQ was read aloud to participants in English or Spanish.

For each DSQ dietary category, we calculated intake amount for all participants with complete data based on recommended scoring procedures using SAS 7.1 software^(16,17)^. We calculated descriptive statistics for socio-demographic variables and dietary intake variables. We defined recommended intake values for fruit, vegetables, fibre, whole grains, dairy products and Ca using the lower limit of values recommended by the 2020–2025 Dietary Guidelines for Americans’ ‘2000-calorie Healthy US-style eating pattern for adults’^(11)^. The recommended intake value for sugar reflects the upper limit of recommended values according to the same guidelines^(11)^. For participants who did not meet the minimum recommended intake of fruit, vegetable, fibre, whole grain, dairy products or Ca, and for participants who exceeded the maximum recommended intake of added sugar, we calculated the difference between recommended and reported intake.

We performed exploratory analysis to investigate associations between socio-demographic variables and dietary intake. After log transformation of the dietary intake variables with non-normal distributions (fruit, vegetables, dairy products, whole grains, Ca and added sugar), we fit bivariate and multivariable linear regressions to investigate associations. Statistical tests were considered significant at *p* < 0·05. Statistical analyses were performed using Stata 16 software^(18)^. This study was approved by the UT Southwestern Medical Center Institutional Review Board (STU-2019-1350).

## Results

The mean age of participants (*n* = 320) was 47 years. Most participants (90%) were female, 44·7% were Black or African American (hereafter Black) and 36·6% were Hispanic or Latino (hereafter Hispanic) (Table 1). Most participants (88·4%) had household incomes below the federal poverty level and 72·8% reported their households had at least one child. Overall, 61·6% of participants were food secure.

**Table 1:** Demographics of participants (*n* = 320)

		Percentage
Age (mean)		46·7
Female		90·0
Race and ethnicity	Black or African American	44·7
	Hispanic or Latino	36·6
	White or Other	18·8
Education	Less than high school graduate	35·9
	High school graduate or GED	35·3
	More than high school/other	28·8
Marital status	Married	29·4
	Single	39·1
	Divorced/other	31·6
Food security status	Food insecure	38·4
	Food secure	61·6
Household income as % of federal poverty level*	0–49%	50·9
	50–74%	20·3
	75–99%	17·2
	100% or higher	11·6
Household size	1	14·1
	2–3	25·9
	4–6	43·1
	7+	16·9
Households with 1 + child ≤ 18 years		72·8
Households with 1 + senior ≥ 60 years		32·5

* Monthly household income is expressed as a fraction of 2019 federal poverty guidelines according to household size. The federal ‘poverty guidelines are updated periodically in the *Federal Registe*r by the U.S. Department of Health and Human Services under the authority of 42 U.S.C. 9902(2)^(13)^’. Those who are below 100% are considered to live below the federal poverty limit.

**Fig. 1:**
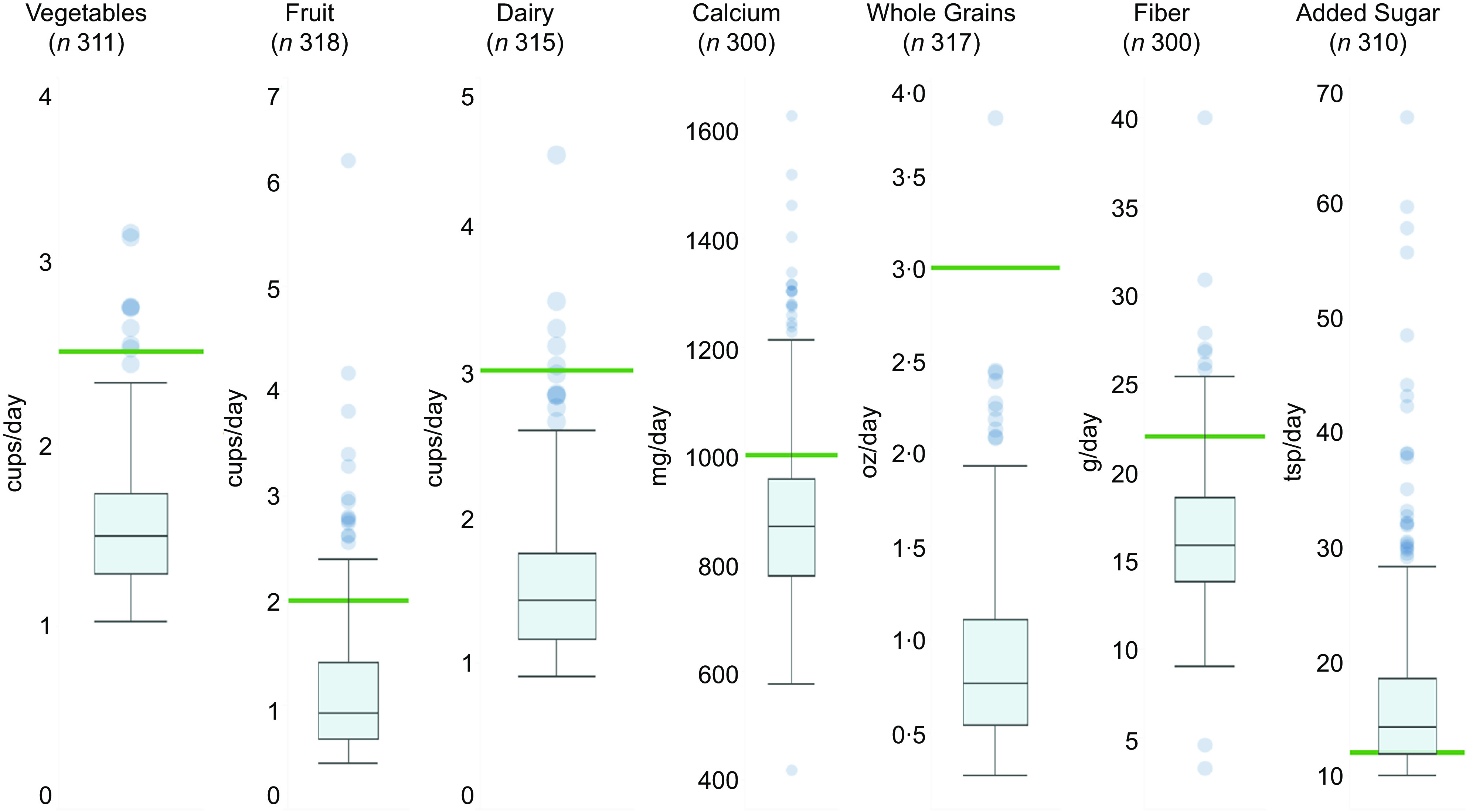
Distribution of daily dietary intake among participants. For each dietary intake category, intake amount was calculated for all participants with complete data. The box and whisker plots illustrate the median, 25^th^ and 75^th^ percentiles, and outliers of dietary intake categories. The green bar represents the minimum recommended intake for vegetables, fruit, fibre, whole grains, dairy products and Ca as well as the maximum recommended intake for added sugar, according to the Dietary Guidelines for Americans, 2020–2025^(11)^.

Dietary intake varied widely across most categories, particularly fruit, fibre, whole grains, Ca and added sugar (Fig. 1). Most participants did not meet the minimum recommended intake values for fruits (88·4%), vegetables (97·4%), fibre (90·7%), whole grains (99·7%), dairy products (98·4%) and Ca (83·4%). Three quarters of participants (73·2%) exceeded the maximum recommended intake for added sugar. No participants met the recommended intake for all seven categories combined.

In bivariate models (online supplementary material, Supplemental Table 1), participants who were food insecure had higher dairy product intake compared with those who were food secure; compared with Hispanic participants, White participants had increased vegetable intake and Black participants had reduced fruit intake. Compared with Hispanic participants, in multivariable models that included all covariates (online supplementary material, Supplemental Tables 2–8), Black participants had reduced whole grain intake (*β* = −0·18, *p* = 0.016) and reduced fruit intake (*β* = −0·18, *p* = 0·021), and White participants had increased vegetable intake (*β* = 0·08, *p* = 0·046). Notably, these differences were relatively small. Black participants consumed an average of 0·82 ounces of whole grains (sd = 0·58) and 1·09 cups of fruit (sd = 0·59) while Hispanic participants consumed an average of 0·9 ounces of whole grains (sd = 0·44) and 1·2 cups of fruit (sd = 0·7). White participants consumed an average of 1·67 cups of vegetables (sd = 0·45) while Hispanic participants consumed an average of 1·52 cups of vegetables (sd = 0·32).

For participants who did not meet the minimum recommended intake of fruit, vegetables, dairy products, fibre, whole grains and Ca, and for participants who exceeded the maximum recommended intake of added sugar, the gaps between the daily recommended intake and daily reported intake of dietary groups are shown in Table 2; for instance, the gap between recommended and reported intake for vegetables was 1·0 cups/d.

**Table 2:** Bridging the gap between recommended and reported intake for dietary categories

	*n*	Recommended daily amount*	Difference between participant intake and recommended intake,† median (IQR)	Healthful food pantry items that could bridge the gap‡
Fruits (cups)	281	≥2 cups	1·0 (0·8–1·3)	1 cup canned pineapple chunks in water or 100% fruit juice
Vegetables (cups)	303	≥2·5 cups	1·0 (0·8–1·2)	1 cup canned mixed vegetables (low Na or no salt added)
Fibre (g)	272	≥22 g	6·6 (4·3–8·5)	½ cup black beans, drained
				1 cup sliced carrots (low Na or no salt added)
				¼ cup dry lentils
Whole grains (oz)	316	≥3 oz	2·3 (1·9–2·5)	1 cup cooked brown rice
				2 slices whole grain bread
Dairy products (cups)	310	≥3 cups	1·6 (1·3–1·8)	1·5 cups milk and/or yogurt
Ca (mg)	247	≥1000 mg	173·4 (100·0–246·6)	1 packet fortified instant oatmeal
				½ cup milk or fortified, unsweetened plant-based milk
Added sugar (tsp)	227	≤12 tsp	4·1 (1·5–8·3)	Replace 1 can of soda with unsweetened beverage or water

* Recommended daily intake values obtained from Dietary Guidelines for Americans, 2020–2025^(11)^.

† The difference between minimum recommended intake and reported intake was calculated for participants who did not meet minimum recommended intake of fruit, vegetable, fibre, whole grain, dairy products or Ca. The difference between maximum recommended intake and reported intake was calculated for participants who exceeded the maximum recommended intake of added sugar.

‡ Examples adapted from recommended healthful foods for food banks to ‘actively seek, procure, and distribute^(26)^’.

## Discussion

Despite receiving both SNAP benefits and food pantry assistance, and despite 62% of our sample being food secure, most participants in our study did not meet dietary intake recommendations. The prevalence of poor dietary and nutrient intake present in our study population is striking. Previous studies report similarly insufficient intake of key food groups and nutrients among clients who use the charitable food system^(5,6)^. Likewise, previous studies of SNAP beneficiaries are consistent with our findings that this group is not meeting micronutrient and macronutrient intake recommendations^(7)^. Our findings extend our understanding of dietary intake in those receiving food assistance by showing that, in a population that is receiving assistance from both charitable and governmental food assistance programmes, substantial gaps in dietary and nutrient intake persist.

While there were some statistically significant differences in dietary intake by socio-economic characteristics, the differences were not substantial. These findings are consistent with prior research in food pantry populations, which have not identified meaningful associations between diet quality and age, race, ethnicity, sex, education or income^(5,6,8,9)^. In addition, we found no association between household size and dietary intake, a finding that has, to our knowledge, not been characterised in previous studies of individuals receiving food assistance. In a national sample of low-income adults, food insecurity was associated with lower dietary quality^(19)^. In our sample of food pantry clients receiving SNAP benefits, food insecurity was not significantly associated with dietary intake categories based on multivariable analysis. Overall, our results suggest that interventions within food assistance programmes to improve diet should focus on all clients, rather than targeting a particular subset of clients.

Although the current food system shapes the poor dietary quality of all Americans^(20)^, food assistance programmes can still work to improve the dietary quality of the millions of individuals who access their programmes each year. In our study, the gaps between daily median intake and daily recommended intake for diet categories could be partially bridged by food obtained within the charitable food system (Table 2). In addition to promoting a healthier diet through improvements in SNAP policy and infrastructure, this is an important implication because food obtained from food pantries is a substantial part of the diet for low-income clients^(3,5)^. Moreover, many clients rely on food pantries for assistance over long-term periods^(3,21)^. Food pantry networks should prioritise distribution of healthier foods like fruits, vegetables (including beans, peas and lentils) and whole grains using evidence-based strategies, such as optimising implementation of organisational nutrition guidelines, adapting choice architecture and improving refrigerator storage^(22–26)^. Additionally, the dietary quality of SNAP participants should be prioritised by optimising fruit and vegetable incentives, increasing retail healthy marketing strategies, strengthening SNAP-Education infrastructure and aligning SNAP with other food and health-related government agencies^(27,28)^.

Our study has several limitations. Our sample included mostly female participants receiving SNAP benefits at two urban, high-capacity, client-choice food pantries in North Texas; therefore, they may not be generalisable to other settings or population groups. The DSQ is a brief nutrition screener; it does not quantitatively assess all dietary and nutrient factors of interest, including processed meat and Na. Additionally, calculations may underestimate dairy products and Ca intake^(13)^. A more accurate measure of diet intake, such as multiple 24-h dietary recalls, may be more likely to detect differences in intake among socio-demographic groups. Additionally, use of a single, validated total score to reflect overall diet quality, such as the Healthy Eating Index^(29)^, may better capture dietary differences among populations.

## Conclusions

Despite receiving assistance from both charitable and governmental food assistance programmes, participants faced substantial gaps in dietary intake. Still, these gaps could be partially bridged with healthy foods obtained from food pantries in addition to improvements in SNAP to promote a healthier diet. Within the charitable food system, multifaceted interventions are needed to modify inventory, incentivise nutritious choices and increase distribution of nutritious foods. For SNAP, priorities include strengthening SNAP-Ed, expanding and optimising fruit and vegetable incentives and retail healthy marketing strategies and improving alignment of SNAP with other federal and state agencies.

## Supplementary material

For supplementary material accompanying this paper visit https://doi.org/10.1017/S136898002200074X. click here to view supplementary material
