# Supplementary material for: Dietary intake of individuals receiving Supplemental Nutrition Assistance Program and food pantry assistance in North Texas
Source: Public Health Nutr. 2022 Mar 24;26(5):1082–7. doi: 10.1017/S136898002200074X (PMC10346013; doi:10.1017/S136898002200074X)
Supplement: Supplementary file 1 [file S136898002200074Xsup001.docx]

**Supplementary Table 1**. Bivariate Regression Results

|  | Fiber | Whole grains | Vegetables | Fruits | Added sugar | Dairy | Calcium |
| --- | --- | --- | --- | --- | --- | --- | --- |
|  | Coefficient (95% confidence interval) | | | | | | |
| Sex |  |  |  |  |  |  |  |
| Male (ref) |  |  |  |  |  |  |  |
| Female | -1.11 (-2.64, .42) | -0.04 (-.23, .14) | -0.07 (-.15, .01) | 0.10 (-.09, .29) | -0.03 (-.16, .11) | 0.01 (-.09, .12) | -0.05 (-.11, .02) |
| Race/Ethnicity |  |  |  |  |  |  |  |
| Hispanic/  Latino (ref) |  |  |  |  |  |  |  |
| Black/  African American | -0.59 (-1.61, .43) | -0.11 (-.23, .02) | -0.02 (-.07, .04) | -0.13 (-.25, -.00)* | -0.06 (-.15, .03) | -0.04 (-.11, .03) | 0.00 (-.04, .05) |
| White/Other | 0.41 (-.90, 1.73) | -0.09 (-.24, .07) | 0.08 (0.01, .15)* | -0.07 (-.22, .09) | 0.02 (-.09, .14) | 0.01 (-.08, .10) | 0.04 (-.01, .10) |
| Education level |  |  |  |  |  |  |  |
| Less than high school (ref) |  |  |  |  |  |  |  |
| High school graduate/GED | -0.33 (-1.41, .76) | -0.03 (-.16, .10) | 0.01 (-.05, .06) | -0.06 (-.19, .07) | -0.03 (-.13, .07) | 0.04 (-.03, .11) | 0.03 (-.02, .08) |
| More than high school/other | 0.19 (-.98, 1.35) | -0.03(-.17, .11) | 0.01 (-.05, .07) | 0.05 (-.09, .19) | -0.03 (-.13, .07) | -0.06 (-.13, .02) | -0.01 (-.06, .04) |
| Marital status |  |  |  |  |  |  |  |
| Married (ref) |  |  |  |  |  |  |  |
| Single | 0.74 (-.38, 1.86) | 0.07 (-.06, .20) | 0.04 (-.02, .10) | 0.04 (-.09, .18) | -0.05 (-.15, .04) | 0.02 (-.06, .09) | 0.04 (-.01, .08) |
| Divorced/  widowed/other | 0.09 (-1.11, 1.28) | -0.04 (-.18, .10) | -0.02 (-.08, .04) | 0.05 (-.09, .19) | -0.05 (-.15, .06) | -0.01 (-.09, .07) | 0.01 (-.04, .06) |
| Age category |  |  |  |  |  |  |  |
| 18-39 (ref) |  |  |  |  |  |  |  |
| 40-59 | -0.02 (-1.04, 1.01) | -0.06 (-.19, .06) | -0.01 (-.06, .05) | 0.01 (-.12, .13) | -0.04 (-.13, .05) | -0.05 (-.12, .02) | -0.01 (-.06, .03) |
| 60+ | -0.60 (-1.87, .68) | 0.01 (-.14, .17) | -0.01 (-.08, .05) | -0.08 (-.23, .08) | -0.04 (-.15, .07) | -0.06 (-.14, .03) | -0.02 (-.07, .04) |
| Poverty income ratio | 0.37 (-.77, 1.51) | 0.09 (-.05, .22) | 0.01 (-.06, .07) | -0.04 (-.18, .09) | -0.06 (-.16, .04) | -0.02 (-.10, .06) | -0.01 (-.06, .04) |
| Household size | -0.12 (-.32, .07) | -0.02 (-.04, .01) | 0.00 (-.01, .01) | 0.01 (-.02, .03) | 0.00 (-.01, .02) | 0.00 (-.01, .02) | 0.00 (-.01, .01) |
| Food security |  |  |  |  |  |  |  |
| Food secure (ref) |  |  |  |  |  |  |  |
| Food insecure | -0.25 (-1.19, .70) | -0.03 (-.14, .08) | -0.02 (-.07, .03) | 0.10 (-.02, .21) | 0.04 (-.04, .13) | 0.07 (.00, .13)* | 0.03 (-.01, .07) |

*, ** indicates statistical significance at the 95% and 99% level, respectively. Whole grain, vegetable, fruit, added sugar, dairy and calcium intake were log transformed due to non-normal distributions.

**Supplementary Table 2.** Multivariable Regression Results for Fiber Intake

| Coefficient (95% confidence interval) | |
| --- | --- |
| Constant | 17.940 (15.403, 20.478)** |
| Sex |  |
| Male (ref) |  |
| Female | -.594 (-2.243, 1.056) |
| Race/Ethnicity |  |
| Hispanic/Latino (ref) |  |
| Black/African American | -1.164 (-2.418, .090) |
| White/Other | -.045 (-1.441, 1.531) |
| Education level |  |
| Less than high school (ref) |  |
| High school graduate/GED | -.394 (-1.576, .787) |
| More than high school/other | -.260 (-.977, 1.496) |
| Marital status |  |
| Married (ref) |  |
| Single | .973 (-.300, 2.243) |
| Divorced/widowed/other | .196 (-1.135, 1.527) |
| Age category |  |
| 18-39 (ref) |  |
| 40-59 | -.230 (-1.318, .857) |
| 60+ | -.904 (-2.460, .651) |
| Poverty income ratio | .517 (-.688, 1.722) |
| Household size | -.189 (-.445, .067) |
| Food security status |  |
| Food secure (ref) |  |
| Food insecure | -.128 (-1.093, .837) |
| R-squared | .046 |
| No. observations | 298 |

*, ** indicates significance at the 95%, and 99% level, respectively.

**Supplementary Table 3.** Multivariable Regression Results for Whole Grain Intake

| Coefficient (95% confidence interval) | |
| --- | --- |
| Constant | -.076 (-.382, .229) |
| Sex |  |
| Male (ref) |  |
| Female | .007 (-.187, .202) |
| Race |  |
| Hispanic/Latino (ref) |  |
| Black/African American | -.184 (-.333, -.035)* |
| White/Other | -.126 (-.303, .051) |
| Education level |  |
| Less than high school (ref) |  |
| High school graduate/GED | -.004 (-.146, .137) |
| More than high school/other | -.008 (-.155, .138) |
| Marital status |  |
| Married (ref) |  |
| Single | .106 (-.046, .259) |
| Divorced/widowed/other | -.062 (-.219, .100) |
| Age category |  |
| 18-39 (ref) |  |
| 40-59 | -.060 (-.192, .071) |
| 60+ | .017 (-.167, .201) |
| Poverty income ratio | .063 (-.077, .203) |
| Household size | -.027 (-.057, .004) |
| Food security status |  |
| Food secure (ref) |  |
| Food insecure | -.034 (-.150, .082) |
| R-squared | .049 |
| No. observations | 315 |

*, ** indicates significance at the 95%, and 99% level, respectively. Whole grain intake was log transformed.

**Supplementary Table 4.** Multivariable Regression Results for Vegetable Intake

| Coefficient (95% confidence interval) | |
| --- | --- |
| Constant | .416 (.280, .553)** |
| Sex |  |
| Male (ref) |  |
| Female | -.046 (-.135, .043) |
| Race/Ethnicity |  |
| Hispanic/Latino (ref) |  |
| Black/African American | -.030 (-.097, .036) |
| White/Other | .081 (.001, .160)* |
| Education level |  |
| Less than high school (ref) |  |
| High school graduate/GED | -.001 (-.064, .061) |
| More than high school/other | .011 (-.054, .077) |
| Marital status |  |
| Married (ref) |  |
| Single | .051 (-.017, .119) |
| Divorced/widowed/other | -.021 (-.092, .050) |
| Age category |  |
| 18-39 (ref) |  |
| 40-59 | -.013 (-.071, .045) |
| 60+ | .011 (-.071, .093) |
| Poverty income ratio | .025 (-.039, .089) |
| Total household size | .003 (-.011, .017) |
| Food security status |  |
| Food secure (ref) |  |
| Food insecure | -.018 (-.069, .034) |
| R-squared | .053 |
| No. observations | 309 |

*, ** indicates significance at the 95% and 99% level, respectively. Vegetable intake was log transformed.

**Supplementary Table 5.** Multivariable Regression Results for Fruit Intake

| Coefficient (95% confidence interval) | |
| --- | --- |
| Constant | -.067 (-.376, .241) |
| Sex |  |
| Male (ref) |  |
| Female | .123 (-.076, .321) |
| Race/Ethnicity |  |
| Hispanic/Latino (ref) |  |
| Black/African American | -.175 (-.323, -.027)* |
| White/Other | -.072 (-.249, .105) |
| Education level |  |
| Less than high school (ref) |  |
| High school graduate/GED | -.048 (-.190, .094) |
| More than high school/other | .104 (-.043, .251) |
| Marital status |  |
| Married (ref) |  |
| Single | .106 (-.046, .258) |
| Divorced/widowed/other | .086 (-.071, .244) |
| Age category |  |
| 18-39 (ref) |  |
| 40-59 | .014 (-.117, .146) |
| 60+ | -.070 (-.252, .112) |
| Poverty income ratio | -.046 (-.186, .094) |
| Total household size | -.006 (-.037, .024) |
| Food security status |  |
| Food secure (ref) |  |
| Food insecure | .120 (.004, .236) |
| R-squared | .053 |
| No. observations | 316 |

*, ** indicates significance at the 95% and 99% level, respectively. Fruit intake was log transformed.

**Supplementary Table 6.** Multivariable Regression Results for Added Sugar Intake

| Coefficient (95% confidence interval) | |
| --- | --- |
| Constant | 2.886 (2.659, 3.113)** |
| Sex |  |
| Male (ref) |  |
| Female | -.015 (-.161, .131) |
| Race/Ethnicity |  |
| Hispanic/Latino (ref) |  |
| Black/African American | -.038 (-.149, .073) |
| White/Other | .043 (-.088, .174) |
| Education level |  |
| Less than high school (ref) |  |
| High school graduate/GED | -.024 (-.129, .081) |
| More than high school/other | -.005 (-.114, .105) |
| Marital status |  |
| Married (ref) |  |
| Single | -.053 (-.166, .060) |
| Divorced/widowed/other | -.052 (-.169, .064) |
| Age category |  |
| 18-39 (ref) |  |
| 40-59 | -.055 (-.152, .043) |
| 60+ | -.033 (-.170, .103) |
| Poverty income ratio | -.076 (-.181, .028) |
| Total household size | -.006 (-.029, .016) |
| Food security status |  |
| Food secure (ref) |  |
| Food insecure | .050 (-.036, .136) |
| R-squared | .027 |
| No. observations | 308 |

*, ** indicates significance at the 95% and 99% level, respectively. Added sugar intake was log transformed.

**Supplementary Table 7.** Multivariable Regression Results for Dairy Intake

| Coefficient (95% confidence interval) | |
| --- | --- |
| Constant | .382 (.207, .557)** |
| Sex |  |
| Male (ref) |  |
| Female | .019 (-.092, .131) |
| Race/Ethnicity |  |
| Hispanic/Latino (ref) |  |
| Black/African American | -.027 (-.113, .059) |
| White/Other | .028 (-.074, .129) |
| Education level |  |
| Less than high school (ref) |  |
| High school graduate/GED | .029 (-.053, .111) |
| More than high school/other | -.045 (-.129, .039) |
| Marital status |  |
| Married (ref) |  |
| Single | .026 (-.062, .113) |
| Divorced/widowed/other | -.002 (-.02, .088) |
| Age category |  |
| 18-39 (ref) |  |
| 40-59 | -.047 (-.123, .028) |
| 60+ | -.055 (-.159, .050) |
| Poverty income ratio | -.009 (-.090, .072) |
| Total household size | -.003 (-.020, .015) |
| Food security status |  |
| Food secure (ref) |  |
| Food insecure | .065 (-.002, .131) |
| R-squared | .043 |
| No. observations | 313 |

*, ** indicates significance at the 95% and 99% level, respectively. Dairy intake was log transformed.

**Supplementary Table 8.** Multivariable Regression Results for Calcium Intake

| Coefficient (95% confidence interval) | |
| --- | --- |
| Constant | 6.774 (6.661, 6.887)** |
| Sex |  |
| Male (ref) |  |
| Female | -.035 (-.109, .038) |
| Race/Ethnicity |  |
| Hispanic/Latino (ref) |  |
| Black/African American | -.003 (-.060, .053) |
| White/Other | .040 (-.026, .107) |
| Education level |  |
| Less than high school (ref) |  |
| High school graduate/GED | .014 (-.038, .067) |
| More than high school/other | -.011 (-.066, .044) |
| Marital status |  |
| Married (ref) |  |
| Single | .035 (-.021, .092) |
| Divorced/widowed/other | .009 (-.051, .068) |
| Age category |  |
| 18-39 (ref) |  |
| 40-59 | -.020 (-.069, .028) |
| 60+ | -.025 (-.094, .045) |
| Poverty income ratio | .004 (-.050, .058) |
| Total household size | .000 (-.011, .012) |
| Food security status |  |
| Food secure (ref) |  |
| Food insecure | .029 (-.014, .072) |
| R-squared | .034 |
| No. observations | 298 |

*, ** indicates significance at the 95% and 99% level, respectively. Calcium intake was log transformed.
